# Multiple Lines of Evidence Support 199 SARS-CoV-2 Positively Selected Amino Acid Sites

**DOI:** 10.3390/ijms25042428

**Published:** 2024-02-19

**Authors:** Pedro Ferreira, Ricardo Soares, Hugo López-Fernández, Noé Vazquez, Miguel Reboiro-Jato, Cristina P. Vieira, Jorge Vieira

**Affiliations:** 1 Instituto de Investigação e Inovação em Saúde (i3S), Universidade do Porto, Rua Alfredo Allen 208, 4200-135 Porto, Portugal; pedro.ferreira@i3s.up.pt (P.F.); ricardo.soares@i3s.up.pt (R.S.); cgvieira@i3s.up.pt (C.P.V.); 2 Instituto de Biologia Molecular e Celular (IBMC), Rua Alfredo Allen 208, 4200-135 Porto, Portugal; 3 School of Medicine and Biomedical Sciences (ICBAS), Porto University, Rua de Jorge Viterbo Ferreira 228, 4050-313 Porto, Portugal; 4 Faculdade de Ciências da Universidade do Porto (FCUP), Rua do Campo Alegre s/n, 4169-007 Porto, Portugal; 5 CINBIO, Department of Computer Science, ESEI—Escuela Superior de Ingeniería Informática, Universidade de Vigo, 32004 Ourense, Spain; hlfernandez@uvigo.gal (H.L.-F.); mrjato@uvigo.gal (M.R.-J.); 6 CINBIO, SING Research Group, Galicia Sur Health Research Institute (IIS Galicia Sur), SERGAS-UVIGO, 36213 Vigo, Spain

**Keywords:** SARS-CoV-2, coronaviruses, positively selected amino acid sites

## Abstract

SARS-CoV-2 amino acid variants that contribute to an increased transmissibility or to host immune system escape are likely to increase in frequency due to positive selection and may be identified using different methods, such as codeML, FEL, FUBAR, and MEME. Nevertheless, when using different methods, the results do not always agree. The sampling scheme used in different studies may partially explain the differences that are found, but there is also the possibility that some of the identified positively selected amino acid sites are false positives. This is especially important in the context of very large-scale projects where hundreds of analyses have been performed for the same protein-coding gene. To account for these issues, in this work, we have identified positively selected amino acid sites in SARS-CoV-2 and 15 other coronavirus species, using both codeML and FUBAR, and compared the location of such sites in the different species. Moreover, we also compared our results to those that are available in the COV2Var database and the frequency of the 10 most frequent variants and predicted protein location to identify those sites that are supported by multiple lines of evidence. Amino acid changes observed at these sites should always be of concern. The information reported for SARS-CoV-2 can also be used to identify variants of concern in other coronaviruses.

## 1. Introduction

Firstly identified in patients with the common cold [1], coronaviruses (family *Coronaviridae*) are able to infect both humans and animals [2]. Of the 46 species in the *Coronaviridae* family, 7 can infect humans, namely, the *α-coronaviruses* HCoV-229E and HCoV-NL63 and the *β-coronaviruses* HCoV-HKU1, HCoV-OC43, severe acute respiratory syndrome coronavirus (SARS-CoV), Middle East respiratory syndrome coronavirus (MERS), and severe acute respiratory syndrome coronavirus 2 (SARS-CoV-2) [3]. HCoVs are responsible for a considerable percentage of yearly common colds [4]. The ones that cause respiratory syndromes became epidemics, with the most recent one, SARS-CoV-2, turning into a pandemic [5].

Coronaviruses are characterized by their large positive-sense RNA genomes that encode three types of proteins: structural, non-structural, and accessory [6]. The structural proteins are the spike (S), nucleocapsid (N), membrane (M), and envelope (E). Out of the structural proteins, the most abundant is the M protein [7]. It presents an N-terminal ectodomain, a transmembrane domain composed of three helices, and a C-terminal endodomain [8]. This protein is crucial for both nucleocapsid inclusion into the virion and for S protein recruitment [9,10]. The S protein is the one responsible for cell tropism and the host range by mediating the fusion of both membranes, the host’s and viral’s [11]. It is present in a homotrimeric form at the viral surface, with each protomer having two subunits, S1 and S2. The former comprises the apex, including the N-terminal domain (NTD) and the C-terminal domain (CTD), with both of them being able to function as the receptor-binding domain (RBD). The S1-NTD usually binds to sialic acid or to host receptor proteins, while the S1-CTD binds to a variety of protein receptors to induce cell entry [12,13]. The S2 is anchored to the viral membrane and coordinates membrane fusion [11,14]. The nucleocapsid (N) protein has three domains, an N-terminal domain, an RNA-binding domain, and a C-terminal domain. It forms homodimers, because the monomers are unstable [15]. Furthermore, while the C-terminal domain is essential for RNA binding, the N-terminal domain is involved in the inhibition of interferon β (IFN-β), a signaling protein that is released in virus-infected cells causing nearby cells to strengthen their antiviral defenses [16,17]. The E protein is the smallest and least well characterized of the four major structural proteins. It may contribute to the host’s cytokine dysregulation leading to disease [18]. Due to their high degree of conservation, structural genes can be recognized even in a distantly related coronavirus [19].

The degree of conservation of non-structural proteins is much lower than that for structural proteins [19]. Therefore, orthologous genes (i.e., genes that have a shared ancestry but, due to speciation events, diverged) are not always easy to recognize in different coronavirus genomes [20]. The SARS-CoV-2 genome encodes 16 non-structural proteins [21]. NSP1 inhibits host translation and degrades mRNAs from the host [22]. Furthermore, two regions located at the C-terminal region of this protein are involved in inhibiting IFN-β and thus antiviral responses [22]. NSP2 binds to prohibitins 1 and 2 [23]; however, its exact functions remain unknown. NSP3 is the largest membrane-bound protein and is composed of several domains, including the papain-like protease domain, responsible for the cleavage of NSP1-3 and a nucleic acid-binding domain. It also acts as a scaffold to form the replication–transcription complex by associating with both host proteins and other non-structural proteins [24,25]. NSP4 has four transmembrane domains. This protein is believed to be involved in anchoring the viral replication–transcription complex in association with NSP3 and NSP6 [26,27]. The main protease, NSP5, active only as a dimer, processes the viral polyproteins to release mature NSP4-16 proteins [28]. The NSP6 of SARS-CoV-2 is a transmembrane protein that is associated with the generation of autophagosomes from the endoplasmic reticulum of the host [29]. NSP7 and -8 form hollow cylindrical hexadecameric complexes, with dimer conformations, with a charge distribution such that the phosphate backbone of nucleic acids can pass through in the absence of electrostatic repulsion [30,31]. Additionally, the NSP7-8 heterodimer helps stabilize the NSP12 (RNA-dependent RNA polymerase), with NSP8 also aiding in the stabilization of the template RNA. Thus, in the presence of the NSP7-8, the NSP12 affinity to RNA and its activity are greatly enhanced [32]. NSP9 forms dimers that bind to ssRNA and is also involved in viral replication [33]. NSP10 is essential for both NSP14 and NSP16 function, forming the mRNA cap methylation complex [34]. The exoribonuclease, NSP14, takes part in the replication–transcription complex (RTC) and is critical for the proofreading process. Nevertheless, the catalytic region for the exoribonuclease collapses in the absence of NSP10. NSP10 is also critical for the function of NSP16, which catalyzes the 5’-methyl capping of the viral mRNA. Indeed, NSP10 is mandatory for NSP16 activity [35]. NSP13 (helicase) plays a role in the unwinding of double-stranded RNA or DNA (dsRNA/DNA) [36]. It also interacts with the NSP7-8-12 complex, two copies on the opposing side of the RNA-binding site [37]. Furthermore, it has been reported that overexpression of the NSP14 protein is linked with the disruption of the levels of IFN-β [38]. NSP15 is a uridine-specific endoribonuclease highly conserved in coronaviruses, that interferes with the innate immune response, acting as an inhibitor of the interferon response [39,40].

Nine accessory proteins (ORFs 3a, 3b, 6, 7a, 7b, 8, 9a, 9b, and 10) that have no homology with other groups of viruses are also encoded by SARS-CoV-2. The accessory genes can be located between structural protein-encoding genes or within them, such as ORFs 9a and 9b, located within the nucleocapsid gene [41].

The origin of *Coronaviridae* capable of infecting humans is still debated, but it is believed that they originated in animals, with *α-coronavirus* having originated in bats and *β-coronavirus* in rodents [42,43]. However, the findings of close relatives in other groups of species hints to a more complex evolutionary history of these viruses prior to human transmission [44].

As expected for a virus that just jumped the species barrier, at the beginning of the pandemic, the SARS-CoV-2 S protein was ill adapted to its human receptor, the angiotensin-converting enzyme 2 (ACE2; [45]). The mutations that first appeared in the SARS-CoV-2 S protein are predicted to have increased the binding affinity to the human ACE2 receptor, and inferred binding affinity can be used as a predictor of transmissibility [45]. Moreover, mutations that are predicted to lead to a better S/ACE2 fit have increased in frequency in the population due to positive selection, because the strains harboring such mutations leave, on average, more descendants than those not harboring such mutations [45]. This can be inferred by looking, in each codon, at the ratio of nonsynonymous substitutions per nonsynonymous site (d_N_) divided by the ratio of synonymous substitutions per synonymous site (d_S_), because, under purifying selection, this ratio is always below one. Purifying selection (the removal of deleterious mutations) must occur with high frequency in functional genes so as to preserve function [46]. It should be noted, however, that not all coronaviruses use the ACE2 receptor for cell entry. For instance, only three out of the seven coronaviruses capable of infecting humans use the ACE2 receptor [47,48,49]. Moreover, unlike the *β-coronaviruses* SARS-CoV and SARS-CoV-2 that use a single large receptor-binding motif (RBM) at the S protein to bind the receptor, the *α-coronavirus* HCoV-NL63 makes use of different RBMs [50]. Efforts to predict the ability to use this receptor have also been made. The S protein hydrophobic pattern described by Carvalho and Alves [51], YGFY, was able to perform this distinction, although with some exceptions. Nevertheless, another S amino acid pattern described in Soares, Vieira, and Vieira [45], CYX(6)YX(3)T[^V] in positions 488-501 of SARS-CoV-2’s RBM, was able to distinguish, with no exceptions, the *β-coronaviruses* that recognize ACE2, from those that do not have this ability. Furthermore, those that use ACE2 share S protein structural similarities. Even though the RBD of SARS-CoV-2 is located at the S1-CTD, its S1-NTD is capable of interacting with other coreceptors, such as AXL, CLEC4G, LDLRAD3, and TMEM30A, in order to ease the viral entry [52,53].

Being able to predict which amino acid sites, genome-wide, when mutated give an advantage to the coronavirus harboring such mutation is thus important for health policy decision makers. For instance, the Centers for Disease Control and Prevention (CDC) created a four-level classification system based on perceived potential threat. From lowest to highest, these classifications are: Variant Being Monitored (VBM), of Interest (VOI), of Concern (VOC), and of High Consequence (VOHC) [54]. To be considered a variant, one or more amino acid mutations are needed to differentiate it from the wild type, as well as from other variants. Furthermore, genetically related variants that had a common ancestor form a lineage. In cases where the genetic material between two variants is combined, we are in the presence of a recombinant [54], which is a major source of diversity in HCoVs [55,56,57,58]. Nevertheless, due to small viral effective population sizes (a consequence of the frequent bottlenecks and intense purifying selection), viruses present low levels of diversity within populations. Differentiation among viral populations is, however, common and correlations with geographical and temporal outbreaks have been observed [59,60].

Positively selected amino acid sites (PSSs), those likely harboring the amino acid variants responsible for increased transmissibility or to host immune system escape and that thus give an advantage to the strains harboring the positively selected amino acid variants over the others not harboring such variants, have been detected from the very start of the SARS-CoV-2 pandemic that started in 2019. For instance, when using FUBAR [61] and 86 SARS-CoV-2 full-length genome sequences, the latest isolated on 1 March 2020 [62], evidence for diversifying selection was obtained at four amino acid sites, located in four genes (*nsp1*, *nsp3*, *nsp6*, and *ORF3a*). Many such studies followed, using different sampling strategies and positive detection algorithms, such as codeML [63], MEME [64], and FEL [65], and recently, the results of a large-scale analysis, using more than 13 million genomes and three positive selection detection algorithms (FUBAR, MEME, and FEL [66]) have been provided in the COV2var database (https://biomedbdc.wchscu.cn/COV2Var/, accessed on 5 December 2023 [66]). These authors looked at all the mutations with a frequency greater than 1% in at least 1 of the 2735 viral lineages and that occur at least twice within that specific lineage, as well as all mutations occurring in at least 2 lineages. These results must, however, be interpreted with caution, because not all PSSs are identified using the three positive selection detection algorithms used and because the same gene has been surveyed multiple times in different lineages, which may result in the detection of false-positive PSSs. Moreover, by looking at variation within SARS-CoV-2 lineages only, the selective value of the amino acid variants that are used to define lineages may not have been considered, unless such amino acid variants are present in lineages that appeared by recombination between two different lineages that differ at that amino acid position. The combination of this information with that of GISAID [67], where the lineage-defining mutations can be found, and the SARS2-Mutant database [68], where the frequency of the top 10 mutations for each gene is listed, facilitates the interpretation of the results.

Here, we have performed a SARS-CoV-2 genome-wide study, using a more classical approach, using both FUBAR [61] and codeML [63] algorithms, and compared our results with those present in the COV2Var database. PSSs that are not listed as such in the COV2Var database have been here identified, and amino acid sites listed as PSSs in the COV2Var database are not here identified as PSSs. Therefore, in order to get further support for those PSSs that were not identified by both studies, we have also inferred the PSSs in 15 non-SARS-CoV-2 coronaviruses to determine which PSSs are located in regions where PSSs are usually detected. Moreover, we have also considered the location of the PSSs on protein structures to determine whether PSSs are located on the predicted protein interfaces. The frequency of the minor allele has also been taken into account when trying to address the nature of the discrepancies. In total, 199 PSSs have been identified as being supported by multiple sources of evidence, highlighting and narrowing the regions for which amino acid variation should raise concern. Hence, this helps with a quicker assessment of risk of newly formed variants by entities such as the CDC, as positive selection is likely associated with features such as higher transmissibility or immune escape. The rationale here used can be applied to other coronavirus species that affect human health or species that are exploited by humans.

## 2. Results

### 2.1. PSS Inferences in SARS-CoV-2

For SARS-CoV-2, only PSSs inferred using both FUBAR [61] and codeML [63], using 100 randomly chosen sets of 30 sequences each (consisting of 6 sequences from each year, from 2019 to 2023), and that are identified in more than 5% of the runs are reported. Therefore, very rare positively selected amino acid variants may have been missed. For the S protein, 52 PSSs have been identified (Supplementary Figures S1 and S2) that correspond to 4.1% of the S protein size. The 52 PSSs are located on the following SARS-CoV-2 domains: 44 (9.9% of the size of the region) in the S1, and of these, 17 in the NTD and 22 in the RBD; and 8 (1.4% of the size of the region) in the S2, of which 1 in the HR1 and 2 in the TM and CP. The observation that most PSSs map to the S1 subunit is not surprising because this is a region that is responsible for the binding to the host receptor and that is recognized by antibodies [11]. All 52 SARS-CoV-2 PSSs are listed in the GISAID database, meaning that they have achieved a frequency higher than 2% in at least one month since their appearance. Moreover, 42.3% of the amino acid sites listed in GISAID for the S protein have been inferred to be a PSS. When considering only the RBD or the NTD, 64.7% and 31.5% of the sites listed in GISAID have been inferred to be PSSs, respectively.

For the SARS-CoV-2 M protein, 15 (corresponding to 6.8% of the protein size) PSSs have been identified, while for the N protein, 17 (corresponding to 4.1% of the protein size) PSSs were found (Supplementary Figures S1 and S2). For the E protein, only one (corresponding to 1.3% of the protein size) PSS was identified (Supplementary Figures S1 and S2). For NSP2, NSP3, and NSP7, no PSSs were identified, while for the other non-structural proteins, between 1 (NSP6) and 10 (NSP1) PSSs were identified (Supplementary Figures S1 and S2). Relative to the protein size, NSP9 is, however, the one with the highest number of PSSs (6.2% of the protein size).

For the accessory proteins, 10, 1, 4, 5, 1, and 2 PSSs were identified for ORF3a, ORF6, ORF7a, ORF7b, ORF8, and ORF10, respectively (Supplementary Figures S1 and S2). Relative to the protein size, this corresponds to between 0.8% (ORF8) and 11.6% (ORF7b) of all the amino acids.

The results here obtained are compared with those in the COV2Var database (http://biomedbdc.wchscu.cn/COV2Var/index/, accessed on 5 December 2023) [66], where the selective value of all the mutations that occur at least twice within a given lineage and have a frequency greater than 1%, or that occur in more than one lineage, have been analyzed, using a total of 2735 viral lineages. The most important features of this database when considering amino acid polymorphism only are summarized in Table 1. For the structural proteins, between 26.6% and 55.1% of the amino acid sites are variable (the average is 36.9%, and the standard deviation 13.1%), while for the non-structural proteins, between 23.7% and 53.9% of the amino acid sites are variable (the average is 35.2%, and the standard deviation 8.9%). For accessory proteins, between 50.8% and 71.9% of the amino acid sites are variable (the average is 64.8% and the standard deviation 7.6%). The higher average for the accessory proteins than for the structural and non-structural genes reflects the smaller degree of conservation of the former when comparing different coronavirus species (see the Introduction).

Depending on the methodology used (FEL or FUBAR), for the structural proteins, on average, between 28.3% (PSS-FEL) and 62.0% (PSS-FUBAR) of the varying amino acid sites are identified as being PSSs (Table 1). For the non-structural proteins, on average, it varies between 20.0% and 45.2%, while for the accessory proteins, on average, it varies between 24.2% and 54.6%. FUBAR has a tendency to identify more PSSs per gene than FEL (Sign test; *p* < 0.00001) but not more than MEME (Sign test; *p* > 0.05). Moreover, MEME also has a tendency to identify more PSSs per gene than FEL (Sign test; *p* < 0.00001) (Table 1). If considering as true PSSs only those identified by all three methods, when using the FEL approach, only 85 out of the 988 PSSs would be false positives (Table 1—PSS-FEL and PSS-3). This is surprising because FUBAR has been reported to have better power than FEL and a very low false-positive rate [61]. Even when looking at PSSs identified by all three methodologies in the S protein (173, Table 1, PSS-3), the number of PSSs is high when comparing with the recently published literature. For instance, Tang et al. [69], using codeML models M7 and M8 (accepting only those sites with a BEB score larger than 0.95 as a PSS) and 50 randomly chosen SARS-CoV-2 genomes, identified only 12 PSSs at the S protein. The difference between the two numbers is partially explained by the size of the sequence dataset, because the 50 randomly chosen genome sequences used by Tang et al. [69] only represent a fraction of all the amino acid polymorphisms assayed in the COV2Var database. Nevertheless, even when comparing with the 52 PSSs identified by us, using multiple medium-sized datasets, where all the years from 2019 to 2023 are equally represented to avoid a bias toward the years where the sequencing effort was heavier, the two numbers are still very different (173 vs. 52). If we consider, however, as true PSSs only those PSSs that were identified in more than 5% of the lineages where they could be assayed, the discrepancy is not so large any longer (70 vs. 52). From this point onwards, only such PSSs will be considered (this dataset from this point onwards is named COV2Var-int-5%) (Table 1).

**Figure 1:**
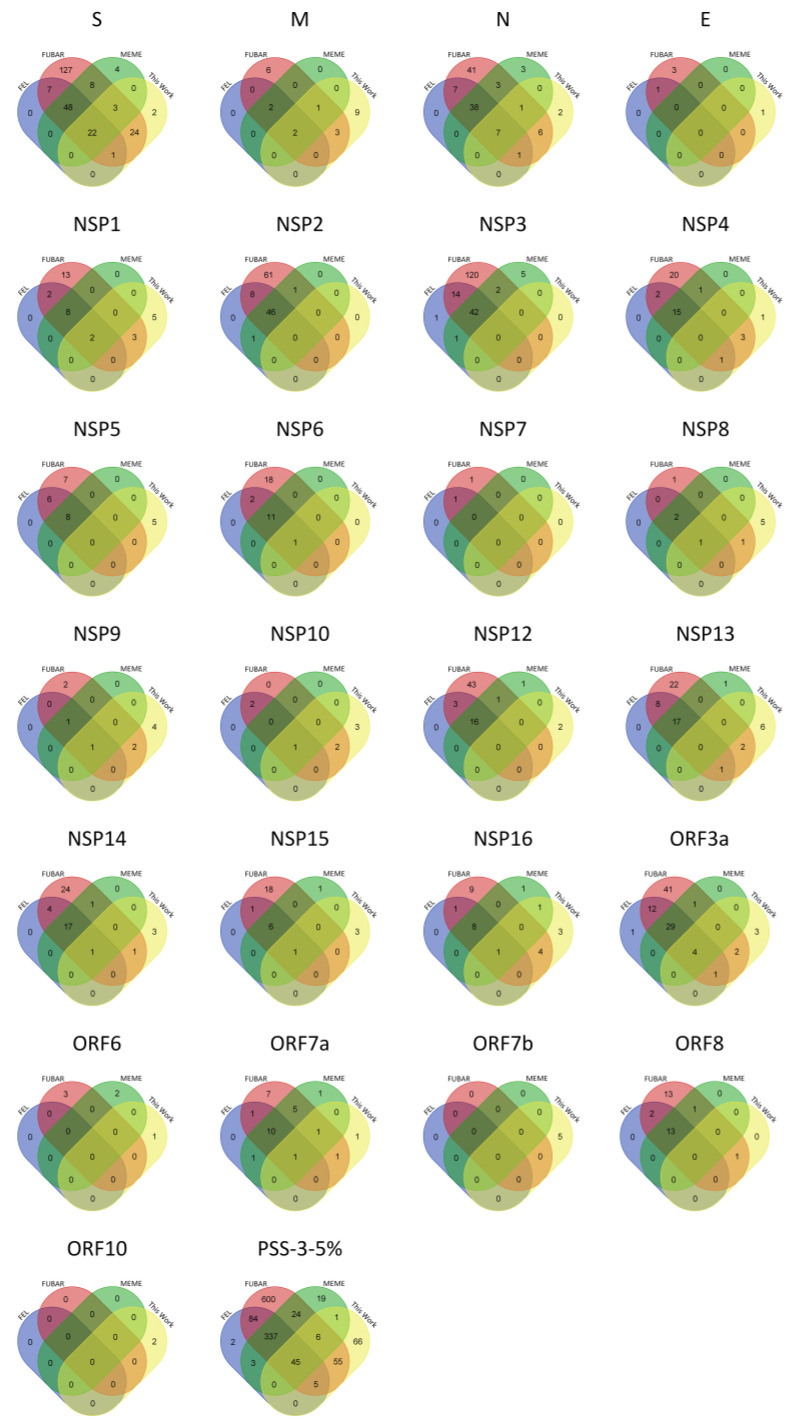
Venn diagrams showing the overlap of the PSSs in the COV2Var database (COV2Var-int-5%) identified by each one of the methods used (FEL (blue), FUBAR (red), and MEME (green)), and those here identified (this work (yellow)), for the structural (S-E), non-structural (nsp1-16), and accessory (ORF3a-ORF10) proteins of SARS-CoV-2.

The comparison of the PSSs identified by us with those from the COV2Var-int-5% dataset revealed a partial overlap only (Figure 1). It is clear that our PSS list is not a subset of those present in the COV2Var-int-5% dataset, because 66 PSSs were only identified by us. In principle, such a discrepancy can be partially explained by the experimental design used to produce the results shown in the COV2Var database. Indeed, Feng et al. [66] only looked at PSSs within viral lineages. Therefore, the selective value of lineage-defining amino acid mutations may not have been assayed, unless they were present as well as polymorphisms within other lineages. This seems to be the case, for instance, of the mutation in site 614 at the S protein.

We also looked at the SARS2-Mutant webpage (http://sars2mutant.com/index; [68]) to obtain the top 10 most frequent mutation sites for all the SARS-CoV-2 proteins analyzed. For the structural proteins S, M, and N, 8, 5, and 7 out of their respective top 10 were here inferred as being under positive selection, while for non-structural and accessory proteins, less than 5 PSSs are among the top 10 most frequent mutation sites (Supplementary Table S1). It should be noted, however, that for NSP7, NSP8, NSP9, and NSP10 the frequency of the top 10 most frequent variants is always below 1%, and that for NSP1, NSP15, ORF6, and ORF7b there is only 1 variant with a frequency higher than 1%.

### 2.2. PSS Inferences in Non-SARS-CoV-2 Coronaviruses

Under the hypothesis that all coronavirus species are under similar selection pressures, independently of using or not the ACE2 receptor for cell entry, further evidence for the selective value of the amino acid changes observed in SARS-CoV-2 may be obtained, by identifying the regions where PSSs are located in non-SARS-CoV-2 coronaviruses, using either FUBAR or codeML. In this case, we use less stringent criteria than in our SARS-CoV-2 analyses (only PSSs identified by both FUBAR and codeML were considered) because for 73.3% of the coronavirus species here analyzed, 60 or fewer sequences were available.

**Figure 2:**
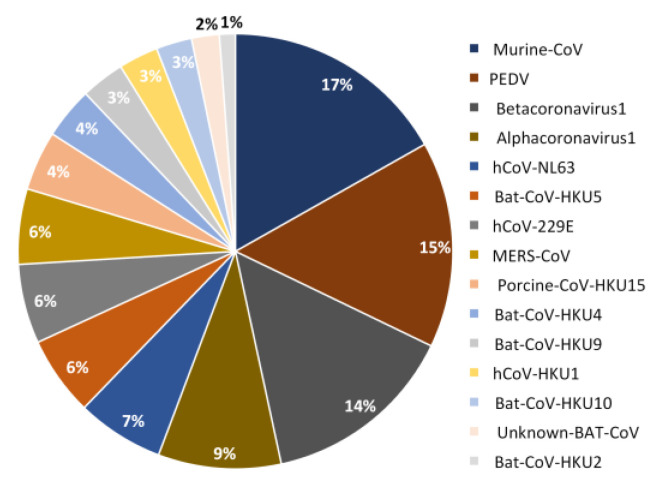
Pie chart showing the contribution (in percentage) of each species to PSSs identified in non-SARS-CoV-2 species.

The PSSs were detected in every non-SARS-CoV-2 species analyzed (15 in total), although 4 species contributed with more than 50% of the total number of PSSs (Figure 2). This is not unexpected, because, if the available sequences are representative of only a specific stage of the evolution of a given coronavirus, they will show only a small fraction of all the mutations that were once involved in the adaptation to the host.

There are 13 PSSs that are identified in more than one species (Table 2), located in both structural and non-structural proteins, showing that, indeed, different coronavirus species are under, at least, partially similar selection pressures.

The total number of different PSSs is 326. Of these, 140 (42.9%) were identified in structural proteins, 139 (42.6%) in non-structural proteins, and 47 (14.4%) in accessory proteins. However, the distribution of these PSSs is non-uniform across the datasets, ranging from 4 in Rhinolophus Bat-COV-HKU2 to 54 in Murine-CoV.

FUBAR inferred 271 PSSs and codeML 106 that correspond to 260 (FUBAR) and 101 (codeML) different amino acid positions in the multispecies protein sequence alignments (Files S1−S4; Table 3). Of these, 35 (33% of the PSSs inferred by codeML) were identified by both codeML and FUBAR.

Across all the datasets, FUBAR was only unable to detect PSSs in NSP9, while codeML failed to detect PSSs in the E protein as well as in eight non-structural proteins (NSP5, NSP6, NSP7, NSP8, NSP9, NSP10, NSP14, and NSP16). Nevertheless, both methods identified the same protein-encoding genes as being of interest, namely, the structural proteins S, M (having very similar results; 50 and 51, 12 and 13, for FUBAR and codeML, respectively) and N (25 and 11 PSSs for FUBAR and codeML, respectively), and NSP3 (58 and 8 PSSs for FUBAR and codeML, respectively). Additionally, FUBAR indicates NSP2 as being of interest, with 15 PSSs; however, codeML only detected 2 (Table 3). The accessory genes could not be compared across species due to the lack of conservation.

When aligning the sequences of the orthologous genes of non-SARS-CoV-2 species and those of SARS-CoV-2, because very divergent species are being compared, only 77.1% (215 out of 279 PSSs) of the PSSs identified in non-SARS-CoV-2 species correspond to non-gapped sites in the corresponding SARS-CoV-2 sequences.

There were 59 S protein PSSs, identified in 13 out of the 15 non-SARS-CoV-2 species analyzed, that mapped on the following SARS-CoV-2 domains: 43 in the S1, and of these, 24 in the NTD and 12 in the RBD; and 16 in the S2, of which 1 in the HR1 and 2 in the TM and CP (Supplementary Figures S1 and S2). The observation that most PSSs map to the S1 subunit is not surprising because, as stated above, this is the region that is responsible for the binding to the hosts receptor and that is recognized by antibodies [11]. Therefore, 4.6% of all the amino acid sites are PSSs (2.8% if considering only FUBAR and 2.9% if considering only codeML). This number increases to 5.4% when considering only the RBD and NTD regions (3.6% if considering only FUBAR and 3.1% if considering only codeML) and 8.2% (4.8% if considering only FUBAR and 5.8% if considering only codeML), respectively. 

GISAID (https://gisaid.org/ assessed on 24 October 2023) reports 123 (9.7%) SARS-CoV-2 S protein amino acid variants that reached a frequency higher than 2% in any given month. The majority (103 out of 123) of such sites are, as expected, located at the S1 subunit of the protein. In total, 8 (6.5%) of these sites (positions 12, 67, 80, 145, 371, 373, 460, and 677) are present in the list of 59 predicted PSSs, based on data for other coronavirus species. Furthermore, 15 other sites directly flank the PSSs here described. Given the difficulty in aligning with confidence highly divergent species, we count those amino acid sites as a correct prediction as well, rising to 18.7% the number of sites present in the list of 59 predicted PSSs, based on data for other coronavirus species. If our criteria are further relaxed, by using a three-amino-acid window flanking each PSS, 50 out of the 123 amino acid variant sites (40.7%) are present in the list of 59 predicted PSSs, based on data for other coronavirus species (Supplementary Figure S2). 

It is conceivable that some of the amino acid sites listed in GISAID have increased in frequency due to hitchhiking with a positively selected mutation. Therefore, we also compared this data with the 52 PSSs identified in the SARS-CoV-2 S protein (see above). Of the GISAID sites here identified as being PSSs, using a three-amino-acid window around the PSSs, 42.3% (22 out of 52) of the SARS-CoV-2 S protein PSSs could have been predicted using data from other coronaviruses. Therefore, the power to predict PSSs in the SARS-CoV-2 S protein increased only slightly (from 40.7% to 42.3%) when restricting the GISAID amino acid sites list to less than half (42.3%) its original size. This observation implies that many amino acid sites present in the GISAID list, for which we could not find evidence that they are PSSs in SARS-CoV-2, can be predicted from data on PSSs from other coronaviruses. This suggests that, despite the lack of evidence, those amino acid sites may also be PSSs.

When looking at the SARS-CoV-2 S RBD, 64.7% (22 out of 34) of the GISAID sites located in that domain have been inferred to be under positive selection. Of these, using a three-amino-acid window around the PSSs, 40.9% (9 out of 22) could have been predicted by looking at data on other coronavirus species. Also, for the NTD, only 31.5% (17 out of 54) of the GISAID sites in that domain were found to be under positive selection; however, 58.8% (10 out of 17) of these could have been predicted by looking at PSS data on other coronaviruses (Table 4).

Of the 11 PSSs at the M protein, coming from 10 out of the 15 non-SARS-CoV-2 species analyzed, 7 (63.6%) mapped to the ectodomain of the M SARS-CoV-2 protein. This is surprising because this region is only 19 amino acids long. In the SARS-CoV-2 M sequence data (Figure 1), 15 amino acid sites were inferred to be PSSs. These are mainly located in the C-terminal domain (5) and ectodomain (4), reinforcing the notion that the M ectodomain is a frequent target of positive selection. In total, 8 out of the 15 SARS-CoV-2 M PSSs (53.3%) could have been predicted, by using data on non-SARS-CoV-2 PSS data, when using a three-amino-acid window around the PSSs (Supplementary Figure S2).

In the case of the N protein, 32 PSSs were found in 12 species of coronaviruses; however, only 23 PSSs from 10 species have homologs in SARS-CoV-2: 2 of them in the N-tail, 6 in the NTD and C-tail, 4 in the linker and 5 in the CTD region, and 6 elsewhere. When looking at SARS-CoV-2 data (see above), 17 PSSs were found in this protein, of which 8 (47.1%) could have been predicted by looking at PSS data on other coronaviruses, when using a three-amino-acid window around the PSSs (Supplementary Figure S2).

Data on non-SARS-CoV-2 non-structural genes can be used to predict 2, 1, and 1 SARS-CoV-2 PSSs at NSP1, NSP8, and NSP15, respectively (Supplementary Figure S2), showing that the nature of PSSs located on different coronavirus species seems to be very different.

We also used the SARS2-Mutant webpage (http://sars2mutant.com/index, accessed on 5 December 2023 [68]) to determine if, for each protein, the 10 most frequent SARS-CoV-2 amino acid variants show signs of positive selection. For the structural proteins S, M, and N, 8, 5, and 7 out of their respective top 10 have been here inferred as being under positive selection, while for the E gene none were found as being positively selected. For the S, M, and N genes, we could have predicted 50.0%, 53.3%, and 47.1%, respectively, of the top 10 amino acid variants, by looking at PSS data on other coronaviruses, when using a three-amino-acid window around the PSSs. For non-structural proteins, 15 out of 140 (10.7%) of the top 10 amino acid variants could have been predicted by looking at PSS data on other coronaviruses.

Orthologies could not be established for the accessory genes; however, it should be noted that PSSs were found at these genes (Table 3).

### 2.3. PSS Location on Protein Structures

**Figure 3:**
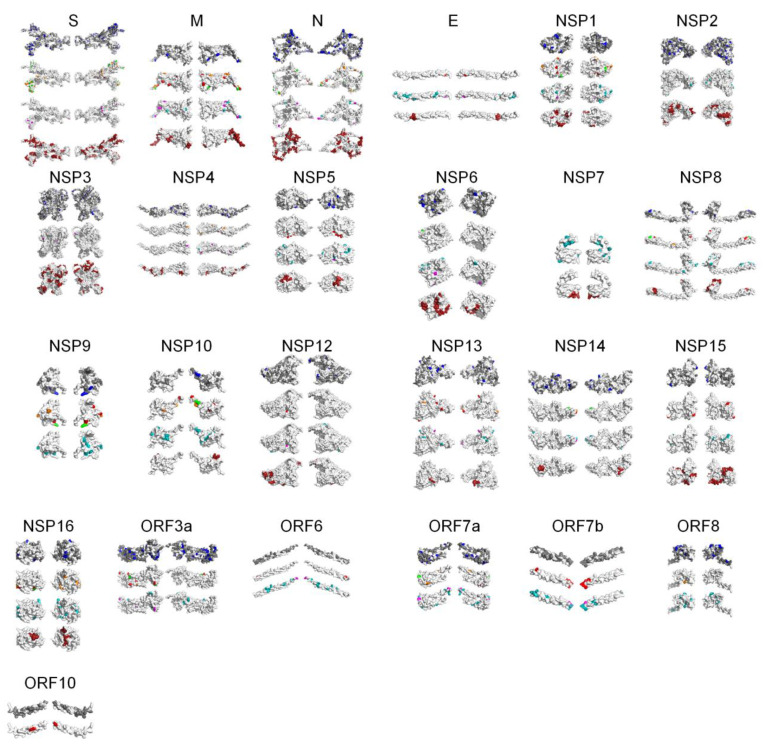
PSS location on SARS-CoV-2 protein monomers. For each protein, from top to bottom: PSSs present in the COV2Var-int-5% list (in blue are PSSs, in gray are variable amino acid sites; no information is available for the E and NSP7 proteins), PSSs identified in this work (green—identified in this work and in the COV2Var-int-5% list; orange—identified in this work and in at least one other method in the COV2Var-int-5%; and red—only identified in this work; there are no PSSs for NSP2, NSP3, and NSP7), the top 10 variants (pink if it has a frequency over 5%, cyan otherwise; there is no information for ORF10), and regions identified as hot PSS regions in non-SARS-CoV-2 species (dark red). For NSP9, as well as for accessory proteins, there is no information for the latter.

**Figure 4:**
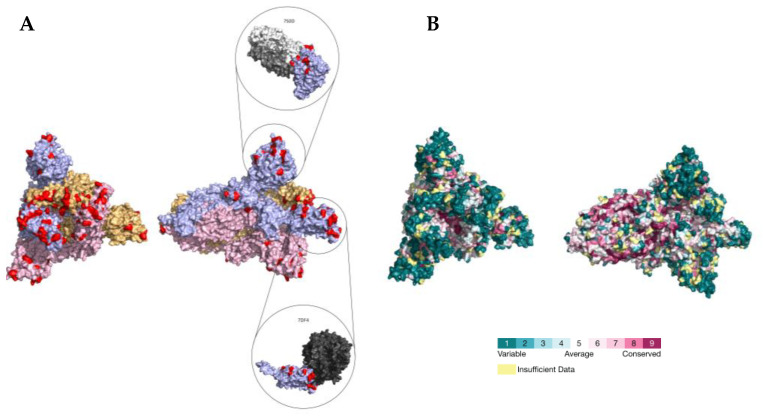
(**A**) Location of PSSs (labeled in red) supported by more than one type of evidence in the SARS-CoV-2 S homotrimer protein structure (PDB accession number 7DF4). Each monomer is shown in a different color. The PDB accession numbers of the docking partners of the S protein are shown above the respective structure (PDB accession numbers 7S0D and 7DF4). (**B**) Consurf projection of the S trimer as in the Consurf Database and its respective color code [70]. The PDB accession number 7DF4 was used as the query.

**Figure 5:**
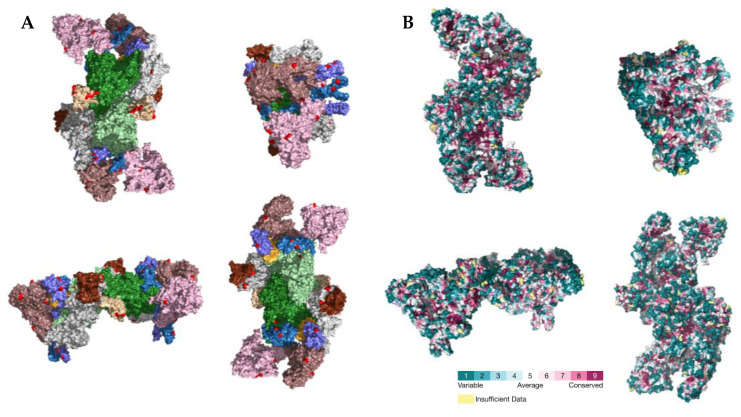
(**A**) Location of PSSs (labeled in red) supported by more than one type of evidence in the SARS-CoV-2 RTC (PDB accession number 7EGQ). NSP7, NSP8, NSP9, NSP10, NSP12, NSP13, and NSP14 are labeled in yellow, violet, beige, brown, green, pink, and white, respectively. (**B**) Consurf projection of the replicase complex dimer as in the Consurf Database [70]. The PDB accession number 7EGQ was used as the query.

The location of the PSSs in the COV2Var-int-5% list, SARS-CoV-2 (this work), the top 10 amino acid variants, and the predictions on where SARS-CoV-2 PSSs are located based on data for other coronaviruses, on the predicted protein structures for every SARS-CoV-2 monomer is shown in Figure 3. It is clear that there is only a partial overlap between the different datasets, and here, we consider that an overlap between two or more of these datasets can be taken as strong evidence for a true PSS (see Supplementary Table S2 for the list of the sites falling into this category). It should be noted, however, that there are amino acid sites identified as PSSs in this work or in the COV2Var database that did not fit the criteria for inclusion in this list but that are located very close to other amino acid sites that were included in this list. As such, they may be also true PSSs. The distribution of the amino acid sites for which there is strong supporting evidence for being a PSS is clearly not random, suggesting that they may have an impact on the protein function. For instance, they may be located at the protein interface with other host and coronavirus proteins. The distribution of sites on the S homotrimer (the functional protein complex; Figure 4) strongly suggests that this is the case, with the majority of sites located at the S protein-binding regions with the ACE2 and neutralizing antibody N-612-014, as well as at the interface between S monomers. For the SARS-CoV-2 RTC, many PSSs are also located at the interface between non-structural proteins (Figure 5).

## 3. Discussion

Amino acid variants that contribute to an increased transmissibility or to host immune system escape are of special concern. Because the coronavirus lineages showing such amino acid variants are likely to increase in frequency due to positive selection, they can be likely identified using different methods, such as FEL, MEME, FUBAR, and codeML. Nevertheless, the results of the use of different methods do not always agree, raising the issue of whether some of the identified PSSs are false positives. This is especially important in the context of very large scale projects where hundreds of analyses may be performed for the same protein-coding gene. In this case, some sort of correction must be performed to account for the multiple tests being performed. When a large number of sequences is available, as is the case for SARS-CoV-2, the sampling scheme may also have an impact on which PSSs are identified. For instance, the results reported in the COV2Var database were obtained by looking at variation within SARS-CoV-2 lineages only. Therefore, the selective value of the amino acid variants that are used to define lineages may not have been considered, unless such amino acid variants are present in lineages that appeared by recombination between two different lineages that differ at that amino acid position. Because of these issues, several lines of evidence must be considered in order to identify true PSSs only.

In this work, we have identified PSSs using both FUBAR [61] and codeML [63] and 100 randomly chosen sets of 30 sequences (each consisting of 6 sequences from each year, from 2019 to 2023). Because, for each gene, 100 independent tests are being performed, only those PSSs that are identified in more than 5% of the runs are trusted. Moreover, only PSSs that are identified by both FUBAR and codeML are considered true PSSs. The comparison of our results with those available in the COV2Var database, even when considering only those sites for which the minor amino acid variant has a frequency equal or greater than 1%, and considering as true PSSs only those sites that were identified in at least 5% of the tests performed for a given gene and method, and that were identified by all three methods used (FEL, MEME, and FUBAR), revealed a partial overlap only. Therefore, these results were reevaluated by adding extra layers of information on the location of PSSs on the protein structures and on PSSs identified in other coronaviruses. Indeed, PSSs that were not identified in all the analyses but that are located in the same protein region where PSSs have been identified in all analyses are likely to be true, especially if they are located in regions known to interact with other proteins. Moreover, if a given region has been identified as harboring PSSs in non-SARS-CoV-2 species, they are also more likely to be true PSSs. Despite the difficulties of aligning highly divergent sequences of different sizes, 13 PSSs were identified in more than one non-SARS-CoV-2 species in the same place in the alignment. It should be noted that in some cases the same PSS has been identified in species from different subgenera. If, in order to account for the uncertainties in the alignment, our criteria for a PSS match in SARS-CoV-2 sequences is relaxed to include a three-amino-acid region flanking the PSS, this number increases to 41. This implies that, despite the high degree of divergence of the species being compared, they are under similar selective pressures and that adaptation occurs through the involvement of a relatively small number of protein regions. This information is also interpreted having taken into account the frequency of the top 10 amino acid variants for each gene using the SARS2-Mutant [68] database. Indeed, very rare variants may not have been identified as PSSs when using our sampling scheme, simply because they were not present in the sample. It should be noted that we are not implying that the PSSs identified in some sampling schemes, when using some methods only, and that are located in protein regions not identified as usually under positive selection are necessarily false positives but simply that at present they are not as well supported as the others for which several layers of information support them as true PSSs.

Data on non-SARS-CoV-2 coronaviruses suggest that all genes, with the exception of nsp9, a gene involved in viral replication and binding to ssRNA [33], may be the subject of positive selection. Nevertheless, here, we found no evidence for PSSs in SARS-CoV-2 NSP2, NSP3, NSP7, NSP12, and NSP15. The different coronaviruses may be, however, at different stages of adaptation to the host, and maybe not all genes are equally important at all stages of adaptation. Although sample size is certainly important, this hypothesis may also help explain the very different number of PSSs identified in non-SARS-CoV-2 coronaviruses. Indeed, it may be difficult to infer PSSs when using only sequences that are representative of the later stages of the evolution of a given coronavirus, because such sequences show only a small fraction of all the amino acid variants that contributed to adaptation to the host. The sequence data that are now available for SARS-CoV-2, covering all steps of its evolution, are thus highly valuable when trying to predict regions and amino acid variants of interest in coronavirus species.

The gene with the highest number of well-supported PSSs (54) is the S gene, which is responsible for cell tropism and host range, because it mediates the fusion of the host’s and viral’s membranes [11], and thus, as expected, is one of the main targets of positive selection.

The N-terminal domain of the N protein is involved in the inhibition of IFN-β, a signaling protein that is released in virus-infected cells causing nearby cells to strengthen their antiviral defenses [16,17], and this may be the reason why a large number of well-supported PSSs (32 sites) have also been identified in the gene coding for the N protein. NSP1, NSP13, and NSP15 are also known to inhibit IFN-β [22,38,71], but the number of well-supported PSSs that are inferred for these genes is low.

The *nsp3* gene is also often a target of positive selection, with 10 well-supported PSSs. NSP3 acts as a scaffold to form the replication–transcription complex by associating with both host proteins and other non-structural proteins [24,25]. Because it interacts with host proteins, it may be a likely selection target.

NSP2, encoded by the second non-structural gene showing most PSSs, binds to prohibitins 1 and 2 [23]. Prohibitins may play a role in the internalization of multiple viruses because they have been identified as the receptors of the Chikungunya [72] and Dengue [73] viruses. The role of prohibitins is, however, unknown in SARS-CoV-2.

Among the accessory proteins, for ORF3a only, a significant number of PSSs have been identified as being well supported (sites).

In conclusion, the integration of different types of analyses, using different coronavirus species, sampling schemes and positive selection detection algorithms, and protein location information, allows the identification of a set of well-supported PSSs in SARS-CoV-2 (199 in total) and identifies protein regions where any amino acid variation is of concern. A similar rationale may be used to identify regions of concern in any other coronavirus for which a large number of sequences is available.

## 4. Materials and Methods

### 4.1. The Sequence Data for SARS-CoV-2

The nucleotide sequence data for SARS-CoV-2 was downloaded from BV-BRC (https://www.bv-brc.org/, accessed on 1 December 2023) for every structural gene and ORF1ab and the accessory genes. In order to identify the different genes encoded by ORF1ab, a *tblastx* was performed using, as the query, nucleotide sequences from the SARS-CoV-2 reference genome (NC_045512.2). At most, 10.000 randomly chosen nucleotide sequences representing different amino acid sequences were retrieved for every gene, from complete genomes, for the years 2020ߝ2023. 

Given the computational limitation of codeML regarding the number of sequences times sequence size that it can process, and the amount of SARS-CoV-2 data available, for each coding region, 100 random datasets consisting of six sequences from each year, from 2019 to 2023, were created using SEDA [74]. Only non-redundant sequences, without ambiguous positions and that are a multiple of three were used. The Docker images available for these programs at the pegi3s Bioinformatic Docker Images project (https://pegi3s.github.io/dockerfiles/, accessed on 12 June 2023 [75]) were used.

### 4.2. Sequence Data for Non-SARS-CoV-2 Coronavirus

For 15 non-SARS-CoV-2 *Coronaviridae* species, all the available nucleotide coding sequences coming from complete genomes were downloaded from BV-BRC (https://www.bv-brc.org/, accessed on 1 December 2023; see Table 5). Using SEDA [74], each coding sequence dataset was filtered for complete sequences, without ambiguous positions or in-frame stop codons, and that are different at the amino acid level. At this point, only datasets having more than four sequences remaining were analyzed. In order to detect PSSs, using the Auto-PSS-Genome pipeline [76], sequences coming from the same genome were collected into the same file.

### 4.3. PSS Detection

For SARS-CoV-2, for each coding gene, 100 files with 30 sequences each, 6 from each year (2019–2023), were used as input in the Integrated Positively Selected Sites Analyses (IPSSA) pipeline that can be used to automatically identify positively selected amino acid sites (PSSs) using three different methods, namely, codeML, omegaMap, and FUBAR [76]. In this work, only the codeML and FUBAR algorithms were used. Briefly, for each dataset, all the available nucleotide sequences were translated and aligned using MUSCLE [77] and the corresponding nucleotide alignment was obtained and analyzed using codeML (models M1a, M2a, M7, and M8 [78]) and FUBAR [61] to infer the PSSs. Bayesian phylogenetic inferences were performed, using MrBayes [79], to obtain the phylogenetic tree that is required by these methods. Only alignment positions with a support value above 3 were used. Two independent runs of 1,000,000 generations with four chains each (one cold and three heated chains) and a burn-in of 25% were used. The implemented model of the sequence evolution was the GTR, allowing for among-site rate variation and a proportion of invariable sites. The third codon positions were allowed to have a gamma distribution shape parameter that was different from that for the first and second codon positions. Trees were sampled every 100th generation. The Docker image available for IPSSA (version 1.2.3) at the pegi3S Bioinformatics Docker Images Project (https://pegi3s.github.io/dockerfiles/, accessed on 12 June 2023 [75]) was used.

The nucleotide coding datasets for 15 non-SARS-CoV-2 *Coronaviridae* species were analyzed using the Auto-PSS-Genome pipeline [76]. Briefly, in the first Auto-PSS-Genome step, orthologous genes are identified using a two-way blast approach. Then, after filtering the sequences to keep only those that are a multiple of three, and do not show open reading frame shifts or ambiguous nucleotide positions, a maximum of 1000 randomly chosen sequences are aligned using ClustalꞶ [80] and PSS-inferred using FUBAR [61]. Running times are in the order of minutes, even for a large number of sequences. For genes where no evidence for positive selection is found, the maximum number of sequences that codeML can handle (it depends on the protein size) is randomly chosen from the filtered dataset (see above), aligned using ClustalꞶ [80], and PSS-inferred using the much slower codeML M1a and M2a models [78]. FastTree [81,82] is used to infer the phylogenetic trees that are required by codeML [78] and FUBAR [61]. A list of genes where suggestive evidence for PSSs is found is then obtained and analyzed using the IPSSA pipeline as described above. A Docker image is available for the Auto-PSS-Genome (version 1.9.0) pipeline at the pegi3S Bioinformatics Docker Images Project (https://pegi3s.github.io/dockerfiles/, accessed on 12 June 2023) [75]).

The results of these analyses, using two different methods of PSS detection (FUBAR [61] and codeML [63]), are publicly available at the B+ database (http://bpositive.i3s.up.pt/, accessed on 1 January 2024).

### 4.4. Orthologous Gene Identification

In order to identify orthologous positions in sequences coming from different coronavirus species, first, using BLAST [83], the sequence orthologies were established. Then, using a reference sequence from each species and gene, the sequences were aligned using ClustalꞶ [80]. Docker images are available for these applications at the pegi3S Bioinformatics Docker Images Project (https://pegi3s.github.io/dockerfiles/, (accessed on 12 June 2023) [75]). The protein alignments used in this work are provided as supplementary material (Supplementary Files 1).

### 4.5. Mapping of PSS on Protein Structure Prediction

The protein 3D models were retrieved from the ColabFold [84] or from the PDB database and PSS-mapped using PyMOL [85].

## Figures and Tables

**Table 1:** Summary of the data present in the COV2Var database regarding amino acid variation and PSSs. Values within brackets are percentages. In the case of variable amino acid sites (VAASs, mutations with a frequency greater than 0.01 in at least one of the 2735 viral lineages and that occur at least twice within that specific lineage, or that are present in at least two different lineages) using the length of the protein, and in the remaining cases using the number of VAASs.

Protein	Length	VAAS	PSS-FEL	PSS-FUBAR	PSS-MEME	PSS-3	#PSS-3-5%
S	1273	481 (37.8)	181 (37.6)	327 (68.0)	352 (73.2)	173 (36.0)	70 (14.6)
E	75	21 (28.0)	1 (4.8)	12 (57.1)	4 (19.0)	1 (4.8)	0 (0.0)
M	222	59 (26.6)	14 (23.7)	30 (50.8)	29 (49.2)	12 (20.3)	4 (6.8)
N	419	231 (55.1)	109 (47.2)	166 (71.9)	179 (77.5)	104 (45.0)	45 (19.5)
NSP1	180	97 (53.9)	43 (44.3)	69 (71.1)	65 (67.0)	33 (34.0)	10 (10.3)
NSP2	638	341 (53.4)	92 (27.0)	183 (53.7)	197 (57.8)	86 (25.2)	46 (13.5)
NSP3	1945	801 (41.2)	138 (17.2)	325 (40.6)	398 (49.7)	118 (14.7)	42 (5.2)
NSP4	500	159 (31.8)	31 (19.5)	74 (46.5)	83 (52.2)	30 (18.9)	15 (9.4)
NSP5	306	83 (27.1)	19 (22.9)	37 (44.6)	42 (50.6)	16 (19.3)	8 (9.6)
NSP6	290	120 (41.4)	24 (20.0)	58 (48.3)	56 (46.7)	24 (20.0)	12 (10.0)
NSP7	83	28 (33.7)	4 (14.3)	9 (32.1)	9 (32.1)	3 (10.7)	0 (0.0)
NSP8	198	60 (30.3)	7 (11.7)	22 (36.7)	13 (21.7)	5 (8.3)	3 (5.0)
NSP9	113	36 (31.9)	8 (22.2)	16 (44.4)	8 (22.2)	5 (13.9)	2 (5.6)
NSP10	139	33 (23.7)	3 (9.1)	13 (39.4)	7 (21.2)	2 (6.1)	1 (3.0)
NSP12	932	256 (27.5)	34 (13.3)	100 (39.1)	94 (36.7)	30 (11.7)	16 (6.3)
NSP13	601	186 (30.9)	42 (22.6)	87 (46.8)	81 (43.5)	36 (19.4)	17 (9.1)
NSP14	527	181 (34.3)	46 (25.4)	91 (50.3)	85 (47.0)	42 (23.2)	18 (9.9)
NSP15	346	125 (36.1)	17 (13.6)	52 (41.6)	55 (44.0)	14 (11.2)	7 (5.6)
NSP16	298	90 (30.2)	15 (16.7)	39 (43.3)	58 (64.4)	15 (16.7)	9 (10.0)
Orf3a	275	183 (66.5)	89 (48.6)	135 (73.8)	144 (78.7)	86 (47.0)	33 (12.0)
Orf6	61	31 (50.8)	7 (22.6)	14 (45.2)	22 (71.0)	6 (19.4)	0 (0.0)
Orf7a	121	86 (71.1)	29 (33.7)	51 (59.3)	82 (95.3)	29 (33.7)	10 (8.3)
Orf7b	43	28 (65.1)	0 (0.0)	0 (0.0)	0 (0.0)	0 (0.0)	0 (0.0)
Orf8	121	87 (71.9)	35 (40.2)	53 (60.9)	72 (82.8)	33 (37.9)	13 (10.7)
Orf10	38	24 (63.2)	0 (0.0)	0 (0.0)	0 (0.0)	0 (0.0)	0 (0.0)
TOTAL		3827	988 (25.8)	1963 (51.3)	2135 (55.8)	903 (23.6)	381 (10.0)

#PSS-3-5%—PSSs that are identified by all three methods, in 5% or more of the analyses that could be performed, and for which the minor variant is above 1% frequency.

**Table 2:** PSSs identified in more than one non-SARS-CoV-2 coronavirus.

Protein		Datasets	SARS-CoV-2 Positions
**S**		PEDV–Betacoronavirus1	75
		PEDV–Alphacoronavirus1	Gap 97-98
**M**		Unknown Bat-CoV–Bat-CoV-HKU9– Bat-CoV-HKU10	4
		Bat-CoV-HKU2–Bat-CoV-HKU9– Bat-CoV-HKU10–Alphacoronavirus1	3
		Bat-CoV-HKU2–Bat-CoV-HKU10	6
**N**		Porcine-CoV-HKU15–Bat-CoV-HKU9	62
		Alphacoronavirus1–hCoV-HKU1	91
		hCoV-HKU1–Murine-CoV	289
**ORF1ab**	**NSP3**	hCoV-HKU1–hCoV-NL63	112
		Murine-CoV–PEDV	162
		Murine-CoV–Betacoronavirus1	1234
	**NSP6**	Murine-CoV–PEDV	138
	**NSP12**	MERS-CoV–PEDV	9

**Table 3:** PSSs identified in non-SARS-CoV-2 coronavirus. PSS Common represents sites identified by both methods. Homologs in SARS-CoV-2 (the PSSs that can be aligned with the SARS-CoV-2 sequences) are shown in brackets. NA—not available.

Protein		PSS-FUBAR	PSS-codeML	PSS Common
**Structural**	S	50 (35)	51 (37)	15 (13)
	M	12 (8)	13 (7)	4 (4)
	N	25 (18)	11 (9)	4 (4)
	E	1 (1)	NA	NA
**Non-Structural**	nsp1	8 (4)	4 (1)	2 (1)
	nsp2	15 (14)	2 (2)	NA
	nsp3	58 (52)	8 (6)	4 (3)
	nsp4	5 (5)	2 (2)	1 (1)
	nsp5	3 (3)	NA	NA
	nsp6	7 (7)	NA	NA
	nsp7	1 (1)	NA	NA
	nsp8	3 (3)	NA	NA
	nsp9	NA	NA	NA
	nsp10	1 (1)	NA	NA
	nsp12	9 (6)	2 (2)	1 (1)
	nsp13	1 (1)	1 (1)	NA
	nsp14	2 (2)	NA	NA
	nsp15	9 (8)	2 (2)	2 (2)
	nsp16	6 (5)	NA	NA
**Accessory**		44	5	2
**TOTAL**		260 (174)	101 (69)	35 (29)

**Table 4:** Distribution of GISAID sites and SARS-CoV-2 PSSs in the spike protein and the respective number of sites predicted by using data from PSSs of other coronaviruses, when considering a three-amino-acid window. Values in brackets are percentages.

	# of Sites (%)
GISAID list	123
Located in RBD	34 (27.6)
Located in NTD	54 (43.9)
SARS-CoV-2 PSSs	52
GISAID sites	52 (100)
Predicted by CoVs PSSs	22 (42.3)
Located in RBD	22
Predicted by CoVs PSSs	9 (40,9)
Located in NTD	17
Predicted by CoVs PSSs	10 (58.8)

**Table 5:** The genome datasets retrieved for non-SARS-CoV-2 Coronaviridae from the BV-BRC database.

Datasets	Genus	Size (Number of Input Files)
Bat-CoV-HKU10	αCoV	8
Rhinolophus-Bat-COV-HKU2	αCoV	8
Pipistrellus-Bat-CoV-HKU5	βCoV	9
Rousettus-Bat-CoV-HKU9	βCoV	10
Tylonycteris-Bat-CoV-HKU4	βCoV	10
Unknown-Bat-CoV	αCoV	19
Murine-CoV	βCoV	29
Human-CoV-HKU1	βCoV	38
Human-CoV-229E	αCoV	44
Human-CoV-NL63	αCoV	58
Porcine-CoV-HKU15	δCoV	60
Alphacoronavirus-1	αCoV	118
MERS-CoV	βCoV	219
Betacoronaviruses-1	βCoV	310
Porcine-epidemic-diarrhea-virus (PEDV)	αCoV	690
*α-coronaviruses*		945
*β-coronaviruses*		625
*δ-coronaviruses*		60

## Data Availability

Data are contained within the article and Supplementary Materials.

## Supplementary Materials

The following supporting information can be downloaded at: https://www.mdpi.com/article/10.3390/ijms25042428/s1.
