# Supplementary material for: Multiple Lines of Evidence Support 199 SARS-CoV-2 Positively Selected Amino Acid Sites
Source: Int J Mol Sci. 2024 Feb 19;25(4):2428. doi: 10.3390/ijms25042428 (PMC10889775; doi:10.3390/ijms25042428)
Supplement: Supplementary file 1 [file ijms-25-02428-s001.zip › Table_S1.pdf]

Supplementary Table S1. PSS summary for the amino acid sites showing the 10 most frequent mutations. No information is available for ORF10 in the SARS2mutant database.

Protein	Position	Frequency	Detection
S	614	97.6	This Work
	484	16.8	FUBAR; This Work
	18	16.2	FUBAR; This Work
	222	15.0	FUBAR; This Work
	501	13.6	FUBAR; This Work
	655	12.8	FUBAR; This Work
	417	12.1	FEL; FUBAR; MEME; This Work
	681	10.7	FUBAR; This Work
	1176	10.7	FUBAR
	138	10.1	FEL; FUBAR; MEME
N	203	86.4	NA
	377	63.3	FEL; FUBAR; MEME; This Work
	63	62.2	FEL; FUBAR; MEME; This Work
	215	56.8	FUBAR; This Work
	204	23.5	This Work
	3	17.2	FUBAR; MEME; This Work
	235	17.3	FUBAR; This Work
	9	4.6	FEL; FUBAR; MEME
	199	2.6	FUBAR; This Work
	220	2.6	NA
M	82	47.2	FUBAR; This Work
	63	24.8	This Work
	19	24.7	FUBAR
	3	16.1	FEL; FUBAR; MEME; This Work
	2	<1	This work
	70	<1	FUBAR; This Work
	81	<1	NA
	28	<1	FUBAR
	30	<1	FEL; FUBAR; MEME
	197	<1	NA
E	9	30.6	This Work
	71	<1	NA
	62	<1	NA
	21	<1	NA
	58	<1	FEL
	73	<1	NA
	55	<1	NA
	68	<1	FEL
	49	<1	NA
	24	<1	FEL; FUBAR
NSP1	135	8.0	This Work
	87	<1	FEL; FUBAR; MEME
	110	<1	FEL; FUBAR; MEME; This Work
	24	<1	FUBAR; This Work
	124	<1	NA
	28	<1	FEL; FUBAR; MEME
	100	<1	This Work
	37	<1	NA
	120	<1	NA
	62	<1	FEL; FUBAR; MEME
NSP2	85	3.9	FUBAR
	81	2.2	FUBAR
	129	1.4	FUBAR
	550	<1	FUBAR; MEME
	339	<1	FEL; FUBAR; MEME
	419	<1	FEL; FUBAR; MEME
	318	<1	FUBAR
	587	<1	FEL; FUBAR
	27	<1	FEL; FUBAR; MEME
	485	<1	FEL; FUBAR; MEME
NSP3	1228	50.0	FEL; FUBAR; MEME
	1469	50.0	FEL; FUBAR
	488	49.5	FUBAR
	183	16.4	NA
	890	16.3	FUBAR
	1412	16.3	FUBAR
	1711	16.3	NA
	489	11.3	NA
	24	11.3	FUBAR

	822	5.2	FUBAR
NSP4	492	70.5	FUBAR; This Work
	167	38.0	FUBAR
	438	10.0	FUBAR; This Work
	264	10.0	This Work
	327	9.6	FUBAR; This Work
	446	4.2	NA
	94	<1	FEL; FUBAR; MEME
	146	<1	NA
	17	<1	NA
	92	<1	FEL; FUBAR; MEME
NSP5	132	30.9	This Work
	90	1.7	FUBAR
	89	1.5	FUBAR
	169	<1	NA
	108	<1	NA
	260	<1	FUBAR
	15	<1	NA
	88	<1	NA
	21	<1	NA
	96	<1	FEL; FUBAR
NSP6	77	71.2	NA
	149	7.7	FUBAR
	181	5.6	FUBAR
	162	3.4	FEL; FUBAR; MEME
	37	3.0	FEL; FUBAR; MEME; This Work
	197	2.5	FEL; FUBAR; MEME
	2	1.2	FEL; FUBAR; MEME
	49	<1	NA
	11	<1	FEL; FUBAR; MEME
	160	<1	FUBAR
NSP7	71	<1	NA
	25	<1	NA
	56	<1	FEL; FUBAR
	77	<1	NA
	75	<1	NA
	3	<1	NA
	26	<1	NA
	81	<1	NA
	63	<1	NA
	58	<1	NA
NSP8	24	<1	FEL; FUBAR; MEME; This Work
	145	<1	This Work
	141	<1	NA
	148	<1	This Work
	187	<1	NA
	123	<1	NA
	10	<1	NA
	89	<1	NA
	34	<1	NA
	198	<1	NA
NSP9	35	<1	This Work
	65	<1	NA
	39	<1	FE; FUBAR; MEME
	42	<1	FUBAR
	101	<1	NA
	24	<1	This Work
	11	<1	NA
	34	<1	This Work
	109	<1	NA
	21	<1	FUBAR; This Work
NSP10	102	<1	NA
	12	<1	NA
	134	<1	FEL; FUBAR; MEME; This Work
	104	<1	FUBAR; This Work
	32	<1	FEL; FUBAR
	51	<1	NA
	111	<1	NA
	84	<1	This Work
	101	<1	NA
	55	<1	NA
NSP12	323	99.3	This Work
	671	42.9	This Work
	838	3.0	FUBAR
	192	2.6	NA
	694	2.0	FEL; FUBAR; MEME
	227	1.9	NA
	875	<1	FEL; FUBAR

	185	<1	NA
	197	<1	FUBAR
	776	<1	NA
NSP13	77	42.6	FUBAR; This Work
	392	9.8	This Work
	334	3.1	FUBAR
	460	2.6	FUBAR; This Work
	164	1.8	FEL; FUBAR; MEME
	341	1.4	FUBAR
	296	1.1	FUBAR
	261	<1	NA
	260	<1	FEL; FUBER; MEME
	36	<1	This Work
NSP14	394	35.2	NA
	42	33.9	This work
	129	1.3	FEL; FUBAR; MEME; This Work
	46	<1	FUBAR
	16	<1	FUBAR
	451	<1	NA
	323	<1	NA
	72	<1	FEL; FUBAR
	43	<1	FUBAR; This Work
	374	<1	FEL; FUBAR; MEME
NSP15	112	9.0	NA
	205	<1	FUBAR
	80	<1	FEL; FUBAR; MEME
	259	<1	This Work
	262	<1	NA
	234	<1	NA
	127	<1	NA
	320	<1	FUBAR
	171	<1	FUBAR
	33	<1	NA
NSP16	216	1.8	FEL; FUBAR; MEME; This Work
	160	1.3	FUBAR
	238	1.0	FEL; FUBAR; MEME
	215	<1	FUBAR; This Work
	35	<1	NA
	179	<1	FEL; FUBAR; MEME
	287	<1	NA
	236	<1	This work
	134	<1	NA
	140	<1	FUBAR; This Work
ORF3a	26	43.2	This Work
	223	10.2	FUBAR; This Work
	57	5.1	This Work
	239	2.8	FUBAR
	106	2.2	FUBAR; This Work
	172	1.8	FEL; MEME
	78	1.5	FEL; FUBAR
	253	1.3	NA
	155	<1	FEL; FUBAR; MEME
	131	<1	NA
ORF6	61	9.5	NA
	48	<1	NA
	46	<1	NA
	57	<1	NA
	21	<1	NA
	33	<1	NA
	27	<1	NA
	20	<1	NA
	49	<1	NA
	24	<1	NA
ORF7a	120	41.4	NA
	82	41.2	FEL; FUBAR; MEME; This Work
	45	1.5	FEL; FUBAR; MEME
	116	<1	FEL; FUBAR; MEME
	71	<1	NA
	118	<1	FEL; FUBAR
	73	<1	NA
	34	<1	FUBAR
	84	<1	NA
	14	<1	NA
ORF7b	40	39.6	This Work
	5	<1	NA
	42	<1	This Work
	43	<1	This Work
	31	<1	NA

	4	<1	This Work
	15	<1	NA
	32	<1	NA
	1	<1	NA
	41	<1	NA
ORF8	24	3.5	NA
	92	2.7	NA
	11	1.7	FUBAR
	65	<1	FEL; FUBAR
	120	<1	NA
	67	<1	FUBAR
	51	<1	FUBAR
	121	<1	FEL; FUBAR; MEME
	100	<1	FEL; FUBAR; MEME
	84	<1	FUBAR
