# Supplementary material for: Multiple Lines of Evidence Support 199 SARS-CoV-2 Positively Selected Amino Acid Sites
Source: Int J Mol Sci. 2024 Feb 19;25(4):2428. doi: 10.3390/ijms25042428 (PMC10889775; doi:10.3390/ijms25042428)
Supplement: Supplementary file 1 [file ijms-25-02428-s001.zip › Table_S2.pdf]

**Supplementary Table S2.** SARS-CoV-2 PSS supported by multiple lines of evidence. For each site, the amino acid of the genome of reference is represented, followed by mutations and the date of their first record, according to COV2var database.

| Protein | PSS | Reference a.a. → mutation(s) (first recording) |
|---|---|---|
| | 5 | L → F (01/2020) |
| | 9 | P → S (03/2020); L (03/2020) |
| | 18 | L → F (01/2020); R (03/2020) |
| | 19 | T → P (04/2020); R (03/2020); I (03/2020) |
| | 67 | A → S (03/2020); V (01/2020) |
| | 70 | V → I (05/2020); F (03/2020) |
| | 77 | K → T (08/2020) |
| | 83 | V → A (05/2020) |
| | 95 | T → A (03/2020); N (04/2020); I (01/2020) |
| | 97 | K → E (04/2020); T (06/2020); R (04/2020); N (03/2020) |
| | 98 | S → P (05/2020); F (01/2020) |
| | 138 | D → H (03/2020); Y (01/2020) |
| | 142 | G → S (03/2020); D (03/2020); V (03/2020) |
| | 145 | Y → N (05/2020); H (03/2020); D (03/2020) |
| | 146 | H → N (04/2020); Y (03/2020); L (04/2020); Q (04/2020) |
| | 147 | K → E (06/2020); T (06/2020); I (05/2020); N (04-10/2020) |
| | 152 | W → R (03-04/2020); L (03/2020); C (01/2020) |
| | 157 | F → S (03/2020); L (02/2020) |
| | 212 | L → I (08/2020); S (04/2020) |
| | 213 | V → L (03/2020); E (11/2020); A (04/2020); G (08/2020) |
| | 222 | A → V (02/2020) |
| | 250 | T → A (03/2020); I (03/2020) |
| | 251 | P → S (04/2020); H (02/2020); L (03/2020) |
| S | 252 | G → S (03/2020); D (05/2020); V (04/2020) |
| | 253 | D → N (04/2020); Y (03/2020); A (03/2020); G (01/2020) |
| | 368 | L → I (05/2020) |
| | 371 | S → P (04/2020); F (04/2020) |
| | 376 | T → A (11/2020) |
| | 385 | T → I (04/2020) |
| | 408 | R → I (01/2020); S (09/2020) |
| | 417 | K → T (04/2020); N (02/2020) |
| | 440 | N → K (03-05/2020) |
| | 444 | K → T (02/2021); R (03/2020); M (01/2021); N (04/2020-01/2021) |
| | 445 | V → L (12/2020); F (04/2020); A (04/2020) |
| | 446 | G → S (04/2020); D (05/2020); V (03/2020) |
| | 452 | L → M (04/2020); Q (03/2020); R (01/2020) |
| | 460 | N → S (04/2020); K (10/2020) |
| | 477 | S → N (01/2020); I (03/2020); R (04/2020) |
| | 484 | E → K (02/2020); Q (03/2020); A (03/2020); G (04/2020) |
| | 486 | F → I (09/2020); L (04/2020); V (07/2020); S (12/2020) |
| | 490 | F → L (03/2020); V (05/2020); S (04/2020) |
| | 493 | Q → R (04/2020); L (05/2020) |
| | 498 | Q → R (04/2020) |
| | 501 | N → Y (02/2020); T (03/2020) |
| | 505 | Y → H (04/2020) |
| | 614 | D → N (03/2020); G (01/2020) |
| | 655 | H → Y (01/2020) |

| | | |
|---|---|---|
| | 658 | N → D (03/2020); S (09/2020) |
| | 680 | S → F (03/2020) |
| | 681 | P → S (03/2020); H (01/2020); R (05/2020); L (03/2020) |
| | 764 | N → K (01/2020) |
| | 950 | D → N (03/2020) |
| | 1147 | S → L (03/2020) |
| | 1219 | G → C (01/2020); V (03/2020) |
| N | 3 | D → H (03/2020); Y (03/2020); V (03/2020); E (03/2020) |
| | 9 | Q → L (04/2020); H (03/2020) |
| | 24 | T →A (10/2020); N (02/2020); I (03/2020) |
| | 25 | G → C (03/2020); D (04/2020); V (03/2020) |
| | 28 | Q → R (03/2020); H (03/2020) |
| | 32 | R → C (03/2020); H (03/2020) |
| | 33 | S → G (01/2020); C (10/2020); N (03/2020); I (03/2020) |
| | 63 | D → N (03/2020); Y (03/2020); G (06/2020) |
| | 67 | P → S (01/2020); L (03/2020) |
| | 90 | A → T (03/2020); S (03/2020); V (04/2020) |
| | 135 | T → A (06/2020); I (03/2020) |
| | 136 | E → D (04/2020) |
| | 178 | G → S (03/2020); V (07/2020) |
| | 199 | P → S (02/2020); L (01/2020) |
| | 202 | S → C (02/2020); N (01/2020); T (02/2020); I (03/2020) |
| | 204 | G → R (01-03/2020); A (04/2020); V (02/2020) |
| | 205 | T → N (05/2020); I (01/2020) |
| | 215 | G → S (02/2020); C (03/2020); V (03/2020) |
| | 218 | A → T (06/2020); S (03/2020); V (03/2020) |
| | 219 | L → F (04/2020) |
| | 235 | S → F (01/2020) |
| | 238 | G → S (04/2020); C (03/2020); D (09/2020); V (03/2020) |
| | 241 | Q → K (05/2020); L (03/2020) |
| | 343 | D →H (03/2020); G (06/2020) |
| | 365 | P → S (02/2020); L (03/2020) |
| | 368 | P → S (03/2020); L (01/2020) |
| | 376 | A → T (02/2020); V (04/2020) |
| | 377 | D → Y (01/2020); A (03/2020); G (03/2020) |
| | 379 | T → A (05/2020); I (02/2020) |
| | 383 | P → S (02/2020); L (02/2020) |
| | 391 | T → I (01/2020) |
| | 413 | S → R (06/2020); I (03/2020) |
| M | 2 | A → T (03/2020); S (01/2020); V (03/2020) |
| | 3 | D → N (05/2020); Y (03/2020); G (02/2020) |
| | 4 | S → F (01/2020) |
| | 6 | G → C (03/2020) |
| | 30 | T → I (04/2020) |
| | 34 | L → F (03/2020) |
| | 63 | A → T (01/2020); S (05/2020) |
| | 69 | A → S (03/2020); V (03/2020) |
| | 70 | V → I (03/2020); L (05/2020); F (03/2020) |
| | 82 | I → V (08/2020); T (03/2020); S (08/2020) |
| | 155 | H → Y (03/2020) |

| | | |
|---|---|---|
| E | 9 | T → A (04/2020); I (03/2020) |
| NSP1 | 24 | R → C (02/2020); L (03/2020) |
| | 28 | V → I (04/2020); L (03/2020) |
| | 62 | P → S (03/2020) |
| | 76 | A → V (04/2020) |
| | 87 | E → D (03/2020) |
| | 100 | S → N (05/2020) |
| | 110 | H → Y (03/2020); R (04/2020) |
| | 135 | S → N (01/2020); R (08/2020) |
| | 137 | G → S (03/2020); C (03/2020); D (03/2020); V (02/2020) |
| NSP2 | 27 | R → L (03/2020) |
| | 31 | A → T (03/2020); V (02/2020) |
| | 36 | S → T (09/2020); Y (08/2020); F (02/2020) |
| | 101 | I → V (03/2020) |
| | 112 | K → N (03/2020) |
| | 164 | C → F (04/2020) |
| | 225 | A → D (06/2020); V (04/2020) |
| | 339 | G → S (01/2020); C (04/2020); D (03/2020) |
| | 419 | A → V (03/2020) |
| | 466 | W → L (04/2020); C (02/2020) |
| | 485 | V → I (03/2020) |
| NSP3 | 10 | D → N (04/2020); Y (04/2020) |
| | 24 | T → I (04/2020) |
| | 149 | A → V (03/2020) |
| | 175 | G → S (02/2020); C (03/2020); D (05/2020) |
| | 183 | T → I (03/2020) |
| | 218 | D → E (02/2020) |
| | 733 | I → V (03/2020); T (04/2020) |
| | 1228 | P → S (03/2020); L (03/2020) |
| | 1250 | T → I (01/2020) |
| | 1412 | I → T (03/2020) |
| NSP4 | 92 | A → V (04/2020) |
| | 94 | V → A (08/2020) |
| | 264 | L → F (02/2020) |
| | 327 | T → A (05/2020); I (12/2019) |
| | 438 | L → I (08/2020); F (01/2020); P (01/2020) |
| | 492 | T → A (04/2020); I (01/2020) |
| NSP5 | 104 | V → I (04/2020) |
| | 132 | P → S (03/2020); H (01/2020); L (03/2020) |
| NSP6 | 2 | A → V (03/2020) |
| | 11 | H → Y (03/2020); Q (04/2020) |
| | 37 | L → F (01/2020) |
| | 162 | I → V (07/2020); T (03/2020) |
| | 182 | V → I (03/2020); F (05/2020) |
| | 195 | E → D (01/2020) |
| | 197 | C → F (04/2020) |
| NSP7 | NA | NA |
| NSP8 | 17 | T → A (03/2020) |
| | 24 | Q → R (04/2020); H (04/2020) |

| | | |
|---|---|---|
| | 145 | T → I (03/2020) |
| | 148 | T → A (03/2020); I (01/2020) |
| NSP9 | 21 | T → N (04/2020); I (03/2020) |
| | 24 | T → I (01/2020) |
| | 34 | T → I (03/2020) |
| | 35 | T → I (03/2020) |
| | 36 | K → R (04/2020); N (01/2020) |
| | 39 | R → K (04/2020) |
| NSP10 | 84 | P → S (03/2020); L (03/2020) |
| | 104 | A → T (06/2020); V (03/2020) |
| | 134 | R → S (03/2020); C (03/2020); H (04/2020); L (03/2020) |
| NSP12 | 323 | P → S (02/2020); L (01/2020) |
| | 671 | G → S (03/2020); C (03/2020); V (04/2020) |
| | 694 | F → Y (06/2020) |
| NSP13 | 36 | S → P (02/2020) |
| | 77 | P → Q (03/2020); L (02/2020) |
| | 164 | H → Y (03/2020) |
| | 260 | D → Y (01/2020) |
| | 392 | R → C (01/2020) |
| | 460 | K → R (03/2020) |
| NSP14 | 42 | I → V (01/2020); L (04/2020) |
| | 43 | P → S (03/2020); L (01/2020) |
| | 129 | N → D (01/2020) |
| | 374 | S → A (03/2020); F (03/2020) |
| NSP15 | 80 | A → V (04/2020) |
| | 259 | K → R (04/2020); N (04/2020) |
| | 282 | D → N (03/2020); Y (04/2020); G (03/2020) |
| NSP16 | 6938 | T → I (03/2020) |
| | 6949 | T → I (02/2020) |
| | 6977 | D → Y (03/2020); G (03/2020) |
| | 7013 | P → T (08/2020); S (05/2020); L (03/2020) |
| | 7014 | R → S (03/2020); C (01/2020); H (03/2020); L (03/2020) |
| | 7034 | P → S (03/2020); L (03/2020) |
| | 7036 | Q → H (03/2020) |
| ORF3a | 26 | S → L (03/2020) |
| | 57 | Q → H (01-03/2020) |
| | 106 | L → F (2/2020) |
| | 108 | L → V (04/2020); F (03/2020) |
| | 110 | A → S (02/2020); V (01/2020) |
| | 155 | D → H (02/2020); Y (02/2020) |
| | 172 | G → R (02/2020); C (03/2020); D (03/2020); V (01/2020) |
| | 223 | T → S (12/2020); N (04/2020); I (02/2020) |
| | 224 | G → R (03/2020); C (02/2020); V (03/2020) |
| | 229 | T → N (10/2020); I (03/2020) |
| ORF6 | NA | NA |
| ORF7a | 45 | P → S (03/2020); L (02/2020) |
| | 82 | V → F (03/2020); A (05/2020) |
| | 116 | L → F (01/2020) |
| | 4 | L → F (03/2020) |

| | | |
|---|---|---|
| ORF7b | 40 | T → I (03/2020) |
| | 42 | H → Y (03/2020) |
| | 43 | A → T (03/2020); S (03/2020); V (01/2020) |
| ORF8 | 84 | L → S (12/2019) |
| | 100 | V → L (01/2020) |
| | 121 | I → L (02/2020); F (03/2020); T (04/2020) |
| ORF10 | NA | NA |
